# Selected salivary parameters in children with idiopathic nephrotic syndrome: a preliminary study

**DOI:** 10.1186/s12903-020-01375-1

**Published:** 2021-01-07

**Authors:** Urszula Kaczmarek, Alina Wrzyszcz-Kowalczyk, Katarzyna Jankowska, Katarzyna Prościak, Monika Mysiak-Dębska, Iwona Przywitowska, Irena Makulska

**Affiliations:** 1 Department of Conservative Dentistry and Pedodontics, Wroclaw Medical University, Krakowska 26, 50-425 Wrocław, Poland; 2 Department and Clinic of Pediatric Nephrology, Wroclaw Medical University, Borowska 213, 50-556 Wrocław, Poland

**Keywords:** Idiopathic nephrotic syndrome, Salivary components, Children

## Abstract

**Background:**

Disturbances in the levels of serum constituents occurring in chronic renal diseases can be reflected in the saliva composition. The aim of this study was to assess some selected salivary components in children suffering from idiopathic steroid-sensitive nephrotic syndrome (iNS).

**Methods:**

A case–control study was performed on iNS and healthy participants. In unstimulated mixed saliva, pH, buffer capacity, total protein, α-amylase, peroxidase, calcium, magnesium, inorganic phosphate, fluoride, urea, uric acid and salivary flow rate were measured. Oral condition was assessed using dmft, DMFT, API and GI indices, usage of fluoride specimens and frequency of tooth brushing. Statistical analysis was performed by Shapiro–Wilk, Brown-Forsythe, Student’s t, ANOVA, Tukey’s and Pearson’s chi-square tests, Pearson’s and Spearman’s correlations, logistic regression and receiver operating characteristic (ROC) curve analysis.

**Results:**

The study involved 94 participants of both genders aged 4–17 (47 cases in relapse or remission phase of iNS and 47 controls) who were treated in the clinic of pediatric nephrology or outpatient dental clinic. Neither group differed in the number of caries-affected primary and permanent teeth, gingival condition or use of fluoride specimens. The iNS group presented lower levels of magnesium (0.41 ± 0.34 vs. 0.60 ± 0.38 mg/dL, P < 0.05) and fluoride (0.15 ± 0.10 vs. 0.21 ± 0.10 ppm, P < 0.01) and higher contents of urea (35.19 ± 15.55 vs. 25.21 ± 10.78 mg/dL, P < 0.01) and uric acid (2.90 ± 1.23 vs. 2.34 ± 1.04 mg/dL, P < 0.05) than the controls. In the iNS participants with relapse, a higher peroxidase activity and lower magnesium content than in the remission phase were found. ROC analysis showed a weak discriminatory power of these salivary constituents for the differentiation of participants with and without disease (accuracy from 66.0 to 67.0%, area under the ROC curve (AUC) from 0.638 to 0.682) and the relapse and remission phases (accuracy 70.2% and 68.1% and AUC 0.717 and 0.675, respectively).

**Conclusions:**

Levels of urea, uric acid, magnesium and fluoride in saliva can be associated with the course of iNS. Salivary levels of peroxidase and magnesium can be related to the phase of the disease. However, the measurements of these parameters cannot be useful as a noninvasive tool in diagnosing iNS and the phase of the disease.

## Background

Nephrotic syndrome is a common chronic glomerular disease occurring in childhood [1, 2]. This disease can be caused by minimal-change nephrotic syndrome, focal segmental glomerulosclerosis, membranous nephropathy, genetic disorders and secondary diseases linked to infections, drugs, and neoplasia; however, the disease can also be idiopathic. The disease involves immune dysregulation, systemic circulating factors, or inherited structural abnormalities of the podocyte [2, 3].

Clinical and biochemical symptoms of the disease result from proteinuria (mostly albuminuria), which exceeds the possibility of compensatory mechanisms in the body. An abnormality of the permselectivity barrier of the glomerular capillary wall, which is unable to restrict the loss of protein, causes the loss of protein with urine. Hypoalbuminemia and hyperlipidemia are found in the blood. Clinical symptoms can include edema of the legs and face, ascites, weight gain, feeling very tired, not feeling hungry, and foamy or bubbly urine [1].

The two types of primary idiopathic nephrotic syndrome are recognized as follows: steroid-sensitive (the most common) and steroid-resistant types. The long-term prognosis for steroid-sensitive cases regarding kidney function is good, but steroid-resistant cases constitute a future risk of chronic or end-stage renal disease. However, even the steroid-sensitive type can have a frequently relapsing course requiring the administration of alternative immunosuppressive agents [1, 2, 4, 5].

Whole saliva is a mixture of the secretion of the major and minor salivary glands, gingival crevicular fluid, mucosal transudate, desquamated epithelial cells, bacteria, viruses and fungi and other cellular components. Some salivary constituents are synthesized by salivary glands, while others originate from the blood through transcellular, passive intracellular diffusion and active transport, paracellular routes by extracellular filtration within the salivary glands or through the gingival crevice [6–8]. Therefore, salivary constituents of serum origin can reflect the abnormalities in the blood composition associated with systemic diseases [9, 10]. In patients with chronic kidney disease (CKD), higher contents of urea, uric acid and creatinine in saliva were found [11–13]. Salivary levels of cortisol, nitrite, uric acid, sodium, chloride, pH, amylase, and lactoferrin have been reported to be markers related to end-stage renal disease [14]. An elevated level of phosphate in saliva was proposed as a biomarker for the initiation of treatment of hyperphosphatemia, which is an important contributor to cardiovascular calcification in chronic renal failure [14, 15]. Therefore, saliva analysis could be used as a noninvasive alternative to plasma analysis for the diagnosis and monitoring of patients with CKD. However, in the case of nephrotic syndrome, it is indefinite whether the abnormality of capillary permeability is restricted to the glomerular capillary wall or can involve other capillaries, including those in the salivary glands.

The study aimed to evaluate selected protein and nonprotein salivary components in children with idiopathic steroid-sensitive nephrotic syndrome compared to healthy controls. We also compared the levels of salivary parameters between the patients in the remission versus relapse phases of the disease. In addition, we assessed the potential usefulness of the studied salivary constituents as markers discriminating diseased and nondiseased children. The null hypothesis has been assumed that there are no differences in the studied salivary constituents between iNS patients and controls.

## Methods

### Study design

The presented case–control study is a part of the project that included clinical examination of oral health parameters, sociodemographic questionnaires and assessment of selected salivary components in idiopathic steroid-sensitive nephrotic syndrome (iNS) patients and controls.

The iNS participants were treated in the Department and Clinic of Pediatric Nephrology, and controls were healthy outpatients attending the dental clinic at the Department of Conservative Dentistry and Pedodontics of Wroclaw Medical University, Poland. The examinations were performed from May 2018 to April 2019. The detailed data on oral health parameters and questionnaire items were published earlier [16]. In this paper, we used only several basic oral health parameters, which could potentially influence the studied salivary components.

The STROBE guidelines for case–control studies were followed [17].

### Participants

The recruited participants (n = 110), both genders, were between the ages of 4 and 17. Nevertheless, 9.1% (n = 10) of the parents refused to consent to the study, and 5.4% (n = 6) of the children refused to be examined. Finally, 94 participants were enrolled in the study. Half of them were patients suffering from iNS and were either in remission (n = 26) or in the relapse phase of the disease (n = 21), being treated in the Clinic of Pediatric Nephrology, and the remaining participants were healthy (n = 47). The inclusion criteria for the iNS patients were the disease diagnosed at least two years earlier than this study and no other acute systemic diseases present at that moment. The iNS phase was recognized by clinical symptoms and laboratory blood tests.

The control group included clinically healthy participants (n = 47) who were outpatients of the dental clinic without a history of impaired renal function or proteinuria or acute or chronic systemic diseases (based on medical interviews of a parent and a child’s health record book) and at the age range and gender corresponding to the iNS patients.

All participants involved in the study had to provide written informed consent from a parent (in addition to a separate written consent from participants age 16 and over), be willing to undergo the oral clinical examination and salivary sample collection, and had to respond to questionnaire items. Participants who did not meet the inclusion criteria were excluded from the study.

### Ethics approval

Approval to conduct the study was obtained from the Bioethics Committee of Wroclaw Medical University (permission no. KB-343/2016). All parents of the recruited participants provided their written consent to participate in the study, and the participants were willing to submit to the investigation. Participation in the study was voluntary and anonymous, and the collected data were treated confidentially.

### Sample size estimation

Sample size determination was based on a t-test for independent groups using a special computer program [18]. The expected difference between means for the two groups for salivary urea content was set at 10.0 (variance equal to 220). The power of the test was set at 90%, and the confidence level was set at 95%. With such assumptions, the required sample size for each group was equal to n = 47.

### Questionnaire items

Data on gender, age, frequency of toothbrushing, use of fluoridated toothpaste and topical professional application of fluoride specimens, and diet were reported by the parents of the participants. The detailed questionnaire items were published earlier [16].

### Oral health status

The clinical oral examination was carried out with the use of artificial light, a plain mirror and a ball-ended dental probe (WHO CPI probe). This process included the assessment of the number of primary and permanent caries-affected teeth (dmft and DMFT values) according to World Health Organization criteria (WHO) [19], oral hygiene using the Approximal Plaque Index—API by Lange et al., 1974 [20] and gingival condition according to the Gingival Index—GI, by Löe and Silness, 1963) [20]. Additional oral health parameters had been analyzed previously [16].

### Salivary sample collection

The mixed saliva was collected after rinsing the mouth with distilled water without external stimulation at least 2 h after breakfast. The child sat with the head bent down and the mouth open and deposited saliva by spitting into a graded test tube that was stored on crushed ice. The salivary flow rate was calculated as ml/min (V) based on the measurement of the volume of the collected saliva sample and the time needed for its collection. The saliva samples were centrifuged for 15 min at a speed of 3500 rpm before the biochemical assays.

### Salivary parameters

The following salivary parameters were assessed: pH (by potentiometric method), buffer capacity (BC, by titration method), total protein (P, by Lowry’s et al. method) [21], α-amylase (Amy, by the assay kit based on Caraway’s method), salivary peroxidase (SPO, by Nbs-SCN^−^ method) [22], calcium (Ca, by the assay kit based on the formation of the chromogenic complex between calcium ions and o-cresolphthalein), magnesium (Mg, by the assay kit based on the reaction of magnesium with xylidyl blue-I), inorganic phosphate (iPh, by the assay kit based on the formation of the chromogenic complex of ammonium molybdate with phosphate), fluoride (F, by the ionic selective electrode Orion 9609), urea (U, by the assay kit based on urease activity), and uric acid (UA, by the assay kit based on uricase activity).

### Assessment of selected components in the blood

Venous blood samples were collected from ill subjects after an overnight fast. After centrifugation, the protein, albumin, urea, uric acid, calcium, magnesium, inorganic phosphate, total cholesterol, triglycerides and creatinine were measured in plasma using an automated standardized multichannel analyzer (Konelab 30i; Thermo Fisher Scientific Inc., bioMerieux, Marcy l’Etoile, France). The estimated glomerular filtration rate (eGFR) was also determined [23]. The blood tests were performed on the same day as salivary sample collection.

### Statistical analysis

The consistency of the obtained results with a normal distribution was verified with the use of the Shapiro–Wilk test and the homogeneity of variance using the Brown–Forsythe test. The quantitative continuous variables with distributions that were not significantly different from normal are presented as the mean and standard deviation, and the qualitative variables are presented as numbers and percentages. Significant between-group differences in the mean values of the variables with a normal distribution were verified using Student’s t-test, ANOVA and Tukey’s test. The association between variables was tested with the chi square Pearson’s test and Spearman’s or Pearson’s correlation coefficients. Logistic bivariate and multivariate regression analyses for dependent variables (presence or absence of the disease) and independent variables (salivary parameters) were carried out. Receiver operating characteristic (ROC) curve analysis was used to evaluate the diagnostic potential of selected salivary constituents to classify the iNS participants compared to controls and the patients with relapse compared to remission. The overall results were assessed by the area under the ROC curve (AUC) and the cutoff values, which were determined based on the best trade-off between sensitivity and specificity. The level of significance was set at P < 0.05. All analyses were computed with Statistica 13 software (PL StatSoft).

## Results

### Distribution of the participants

Ninety-four participants were included in the study; half of them suffered from idiopathic steroid-sensitive nephrotic syndrome, and the remaining participants were healthy. The first episode of the disease occurred between the ages of 4 and 15 (mean 3.8 ± 3.0 years old). The duration of the disease at the time of the examination ranged from 2 to 15 years (mean 6.4 ± 4.0 years), and the number of relapses ranged from 1 to 15 (mean 3.8 ± 2.9).

The mean age of the iNS patients was 9.6 ± 3.9 years and that of healthy participants was 10.8 ± 3.7 years and did not vary substantially (P > 0.05).

The iNS patients at the time of the examination were taking various medications depending on their condition, and they were on diets containing low salt, fat, and cholesterol and rich in protein, fruits, and vegetables, unlike the controls.

### Oral health parameters

The iNS patients and healthy participants did not differ significantly with regard to the number of caries-affected primary and permanent teeth (dmft + DMFT) and components (dt + DT, mt + MT and ft + FT), API value and GI score, usage of fluoridated dentifrice and topical professional application of fluoride specimens. However, the iNS group showed a significantly lower mean number of filled primary and/or permanent teeth (P < 0.001) and less frequent tooth brushing, that is, less than twice a day (P < 0.05) (Table 1).

**Table 1:** Characteristics of the studied participants

		iNS		Control		P-value
		n	%	n	%	
Gender	Male	25	53.2	18	38.3	0.147
	Female	22	46.8	29	61.7	
Age (years)	Mean ± SD	9.6 ± 3.9		10.8 ± 3.7		0.142
Number of caries-affected primary and/or permanent teeth (dmft + DMFT)	Mean ± SD	4.6 ± 3.5		6.0 ± 4.1		0.126
Primary and/or permanent decayed teeth (dt + DT)	Mean ± SD	3.5 ± 3.2		2.4 ± 2.4		0.095
Primary and/or permanent missing teeth (mt + MT)	Mean ± SD	0.02 ± 0.1		0.2 ± 0.8		0.543
Primary and/or permanent filled teeth (ft + FT)	Mean ± SD	1.1 ± 1.6		3.5 ± 3.6		< 0.001**
API (%)	Mean ± SD	54.0 ± 35.7		43.4 ± 27.6		0.108
GI	Mean ± SD	0.7 ± 1.0		0.3 ± 0.6		0.050
Tooth brushing	Twice a day	37	78.7	44	93.6	0.026*
	Once a day or every few days	10	21.3	3	6.4	
Dentifrice	Fluoridated	43	91.5	42	89.4	0.603
	No fluoride	4	8.5	5	10.6	
Topical professional application of fluoride specimens	Yes	16	34.0	17	36.2	0.541
	No or does not know	31	66.0	30	63.8	

*iNS* Idiopathic nephrotic syndrome, *dmft* number of decayed, missing due to caries and filled primary teeth, *DMFT* number of decayed, missing due to caries and filled permanent teeth, *dt* number of decayed primary teeth, *DT* number of decayed permanent teeth, *mt* number of missing primary teeth, *MT* number of missing permanent teeth, *ft* number of filled primary teeth, *FT* number of filled permanent teeth, *API* approximal plaque index, *GI* gingival index, *P-value* level of statistical significance

*Significant difference at P < 0.05

**Significant difference at P < 0.001

### Salivary parameters

**Fig. 1:**
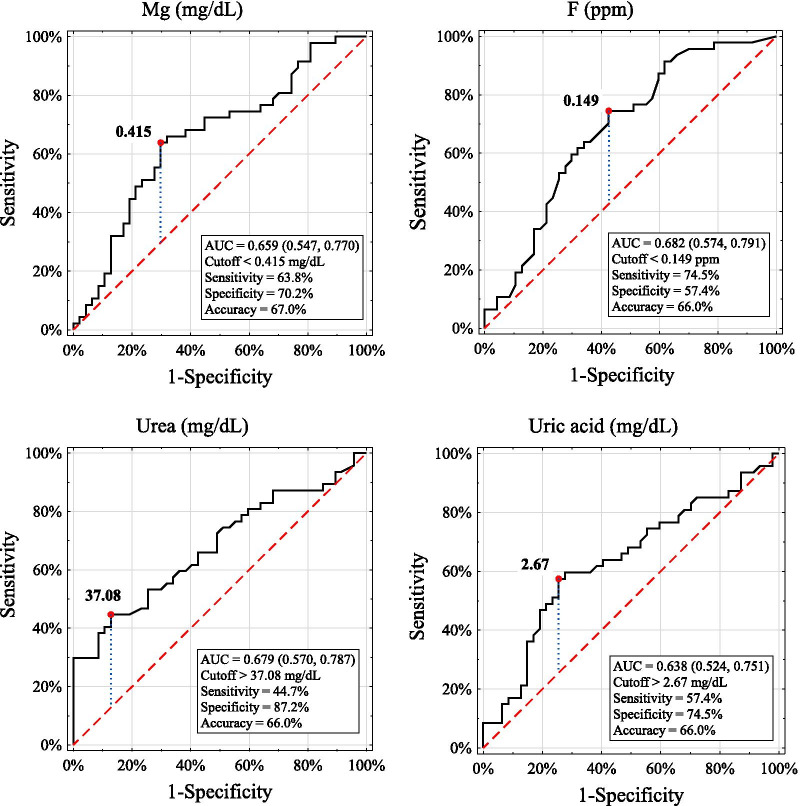
ROC curves and cutoff values for salivary parameters those showed significant difference between the iNS patients and controls. *Mg* magnesium, *ROC* receiver operating characteristic, *AUC* the area under the ROC curve

**Fig. 2:**
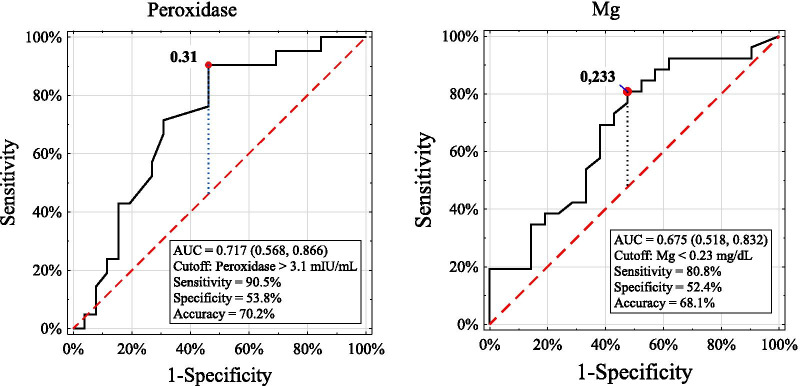
ROC curves and cutoff values for salivary parameters those showed significant difference between the iNS patients with remission and relapse. *Mg* magnesium, *ROC* receiver operating characteristic, *AUC* the area under the ROC curve

The participants suffering from iNS revealed significantly lower concentrations of magnesium (P < 0.05) and fluoride (P < 0.01) and higher urea (P < 0.01) and uric acid (P < 0.05) compared with the controls. The patients in the relapse phase presented a higher SPO level and lower magnesium content than those in the remission phase of the disease, and both subgroups showed a lower content of fluoride and higher urea and uric acid levels than the controls (Table 2). Moreover, the patients in the relapse phase revealed a higher SPO level and a lower concentration of magnesium than the healthy participants. Bivariate logistic regression showed that out of all analyzed salivary variables considered separately, only levels of salivary urea, uric acid, magnesium and fluoride were associated with the disease. In the multivariate regression model (where salivary variables were considered together as independent variables), the levels of salivary urea and magnesium revealed significant associations with the presence of the disease (Table 3). However, ROC curve analysis indicated that the salivary levels of magnesium, fluoride, urea and uric acid were weak classifiers to discriminate the diseased and nondiseased participants. The accuracy for these variables ranged from 66.0 to 67.0%, and the AUC ranged from 0.638 to 0.682 (Fig. 1). Similarly, the salivary levels of SPO and magnesium turned out to be weak markers to distinguish the participants with relapse from the remission phase of the disease because the accuracy was 70.2% and 68.1% and the AUC was 0.717 and 0.675, respectively (Fig. 2).

**Table 2:** Comparison of salivary parameters between the iNS and control participants

Studied groups	iNS			Control n = 47
	Remission n = 26	Relapse n = 21	Total n = 47	
Salivary parameters	Mean ± SD	Mean ± SD	Mean ± SD	Mean ± SD
Flow rate (ml/min)	0.41 ± 0.26	0.48 ± 0.30	0.44 ± 0.27	0.46 ± 0.19
pH	7.27 ± 0.71	7.28 ± 0.70	7.28 ± 0.70	7.50 ± 0.50
Buffer capacity (mmol/L)	6.67 ± 5.83	4.95 ± 2.87	5.90 ± 4.78	4.56 ± 2.45
Total protein (mg/ml)	1.09 ± 0.62	1.24 ± 0.47	1.16 ± 0.56	1.00 ± 0.47
α-amylase (J/ml)	114.7 ± 135.1	95.4 ± 36.6	106.10 ± 103.00	113.36 ± 82.00
Peroxidase (mIU/ml)	0.45 ± 0.50^e^	0.66 ± 0.44^e,g^	0.54 ± 0.48	0.46 ± 0.31^ g^
Calcium (mg/dL)	3.27 ± 1.52	2.93 ± 1.7	3.12 ± 1.45	3.38 ± 2.15
Magnesium (mg/dL)	0.50 ± 0.38^f^	0.31 ± 0.26^f,h^	0.41 ± 0.34^a^	0.60 ± 0.38^a,h^
Inorganic phosphate (mg/dL)	10.34 ± 3.68	11.71 ± 3.50	10.95 ± 3.63	10.33 ± 3.86
Fluoride (ppm)	0.15 ± 0.11^i^	0.15 ± 0.09^ k^	0.15 ± 0.10^b^	0.21 ± 0.10^b,i,k^
Urea (mg/dL)	33.57 ± 16.36^ l^	37.18 ± 14.64^ m^	35.19 ± 15.55^c^	25.21 ± 10.78^c,l,m,^
Uric acid (mg/dL)	2.64 ± 1.09^n^	3.24 ± 1.34^o^	2.90 ± 1.23^d^	2.34 ± 1.04^d,n,o^

*iNS* Idiopathic nephrotic syndrome

Significant difference at P < 0.05 between a–a, d–d, f–f, g–g, n–n and o–o

Significant difference at P < 0.01 between b–b, c–c, e–e, h–h, i–i, k–k, l–l and m–m

**Table 3:** Logistic regression analysis for the presence or absence of the disease as dependent variable

Salivary parameters (independent variables included into regression model)	Results of bivariate logistic regression for dependent variable presence or absence of the disease			Results of multivariate logistic regression for dependent variable: presence or absence of the disease		
	b	OR	p	b	OR	P-value
V	− 0.304	0.738	0.730	1.348	0.299	0.366
pH	− 0.628	0.533	0.092	1.832	0.605	0.385
BC	0.121	1.128	0.114	3.148	1.147	0.060
P	0.632	1.882	0.137	8.278	2.114	0.135
Amy	− 0.001	0.999	0.704	2.710	0.997	0.261
SPO	0.499	1.647	0.347	30.662	3.423	0.106
Ca	− 0.079	0.924	0.495	3.540	1.264	0.213
Mg	− 1.449	0.235	0.021*	1.098	0.093	0.013*
iPh	0.046	1.047	0.416	2.512	0.921	0.352
F	− 6.024	0.002	0.009**	1.006	0.006	0.082
U	0.056	1.058	0.001***	2.912	1.069	0.004**
UA	0.441	1.554	0.022*	4.755	1.559	0.127

*b* logistic regression coefficient, *OR* odds ratio, *V* salivary flow rate, *BC* buffer capacity, *P* total protein, *Amy* α-amylase, *SPO* peroxidase, *Ca* calcium, *Mg* magnesium, *iPh* inorganic phosphate, *F* fluoride, *U* urea, UA *uric acid*, *P-value* level of statistical significance

*Significant effect at P < 0.05

**Significant effect at P < 0.01

***Significant effect at P < 0.001

### Relationship of oral health parameters and salivary constituents

We did not find any significant correlation between oral health parameters and levels of the studied salivary components in either group of participants.

### Serum parameters

The iNS patients demonstrated considerably higher mean levels of total cholesterol and triglycerides in the plasma than the reference range. Several individuals revealed concentrations of protein, uric acid, urea and inorganic phosphate, total cholesterol, triglycerides, creatinine and eGFR values that exceeded the upper limit of the reference ranges. In the relapse phase, compared with the remission phase of the disease, lower concentrations of protein, albumin, and calcium and higher contents of total cholesterol and triglycerides were found (Table 4).

**Table 4:** Blood laboratory data in children with remission and relapse of the disease

Blood parameters	Idiopathic nephrotic syndrome participants			
	Remission n = 26	Relapse n = 21	Total n = 47	Reference range
	Mean ± SD (min–max)	Mean ± SD (min–max)	Mean ± SD (min–max)	
Protein (g/dL)	6.74 ± 0.95^a^ (4.0–8.2)^f^	4.96 ± 0.81^a^ (3.5–6.7)	5.94 ± 1.26 (3.5–8.2)^f^	5.7–8
Albumin (g/dL)	4.14 ± 0.76^b^ (2.1–5.1)	2.55 ± 0.64^b^ (1.4–3.6)	3.43 ± 1.06 (1.4–5.1)	3.5–5.2
Urea (mg/dL)	26.3 ± 7.2 (11–44)^f^	29.8 ± 15.0 (10–72)^f^	27.9 ± 11.3 (10–72)^f^	11–43
Uric acid (mg/dL)	4.86 ± 1.24 (2.7–7.9)^f^	5.43 ± 1.84 (3.5–10.7)^f^	5.11 ± 1.55 (2.7–10.7)^f^	Girls: 2.6–6.0 Boys: 3.5–7.2
Calcium (mg/dL)	9.74 ± 0.60^c^ (8.3–10.6)	9.13 ± 0.84^c^ (7.4–10.4)	9.47 ± 0.77 (7.4–10.6)	8.8–10.8
Magnesium (mg/dL)	1.97 ± 0.21 (1.6–2.6)	2.05 ± 0.19 (1.6–2.3)	2.01 ± 0.21 (1.6–2.6)	1.45–2.6
Inorganic phosphate (mg/dL)	4.75 ± 0.64 (3.5–5.8)^f^	4.79 ± 0.94 (3.2–7.7)^f^	4.77 ± 0.77 (3.2–7.7)^f^	2.5–5
Total cholesterol (mg/dL)	206 ± 63^d^ (122–347)^f^	387 ± 108^d^ (240–659)^f^	285 ± 124 (122–659)^f^	< 170
Triglycerides (mg/dL)	134 ± 125^e^ (40–677)^f^	276 ± 190^e^ (135–969)^f^	199 ± 172 (40–969)^f^	2–9 years < 75 10–17 years < 90
Creatinine (mg/dL)	0.59 ± 0.17 (0.32–1.09)^f^	0.64 ± 0.12 (0.67–0.92)	0.62 ± 0.15 (0.32–1.09)^f^	0.3–1.0
eGFR (ml/min/1.73m^2^)	128.02 ± 18.42 (98.25–160.6)^f^	121.11 ± 21.16 (87.28–187.69)^f^	124.19 ± 20.32 (87.28–187.69)^f^	2–12 years 140 ± 30 13–21 years 126 ± 22

*iNS* Idiopathic nephrotic syndrome, *eGFR* estimated Glomerular Filtration Rate

Significant difference at P < 0.01 between c–c

Significant difference at P < 0.001 between a–a, b–b, d–d, e–e

^f^Individual values over reference range

### Relationship of studied constituents in blood and saliva

We did not find any significant correlation between the same parameters in saliva and blood in the iNS participants, except for a trend of a positive significant correlation of uric acid (r = 0.377, P = 0.051) in patients in the remission phase of the disease (Table 5).

**Table 5:** Pearson correlation coefficients of the studied constituents in blood and saliva in iNS participants

Parameter	Remission n = 26	Relapse n = 21	Total n = 47
P	r = -0.007	r = -0.102	r = -0.125
	p = 0.974	p = 0.659	p = 0.402
Ca	r = 0.196	r = -0.262	r = 0.017
	p = 0.338	p = 0.251	p = 0.911
Mg	r = -0.189	r = -0.261	r = -0.245
	p = 0.356	p = 0.267	p = 0.101
iPh	r = 0.075	r = -0.082	r = -0.003
	p = 0.717	p = 0.733	p = 0.986
U	r = -0.207	r = -0.203	r = -0.167
	p = 0.310	p = 0.377	p = 0.261
UA	r = 0.377	r = -0.153	r = 0.105
	p = 0.051^o^	p = 0.508	p = 0.482

*iNS* Idiopathic nephrotic syndrome, *P* total protein, *Ca* calcium, *Mg* magnesium, *iPh* inorganic phosphate, *U* urea, *UA* uric acid, *r* Pearson correlation coefficient, *p* level of statistical significance

^o^A trend to significant difference at P < 0.05

## Discussion

This preliminary study evaluated selected salivary components in pediatric patients with idiopathic steroid-sensitive nephrotic syndrome. The null hypothesis was rejected, as there were differences in some studied salivary components between the iNS and healthy participants. We demonstrated that the disease is associated with significantly lower magnesium and fluoride concentrations and higher urea and uric acid contents in unstimulated mixed saliva.

iNS is characterized by complex abnormalities in blood tests and treatment by steroids, immunosuppressants or cytostatics, and dietary regimens, which may be reflected in the saliva composition. Unfortunately, we could not compare our results with those of others because data in the literature are scarce and concern the excretion of endogenous proteins (albumin, transferrin, IgG1, and IgG4) [24], salivary cortisol concentration [25] and salivary cytokines [26].

Salivary gland secretion in unstimulated conditions can be reflected by the measurements of flow rate, total protein concentration, and salivary α-amylase activity to some extent. Salivary α-amylase, which is produced by the salivary glands, is the main protein in the saliva and constitutes up to 50% of the total salivary protein [27]. However, we did not observe any significant differences in these parameters between the iNS patients and the controls. Therefore, undisturbed salivary gland activity could be suggested. Polak et al. [26] found a lower total protein concentration in the saliva of children with steroid-sensitive nephrotic syndrome than in the control group.

The salivary buffer capacity acts as an important factor in controlling the pH of the oral environment. The buffer capacity of saliva involves three major systems: carbonic acid/bicarbonate, phosphate, and protein [28]. We did not find any significant difference in the levels of pH, buffer capacity, or inorganic phosphate between the iNS and healthy participants.

Several studies have emphasized the pathophysiological importance of oxidative stress in patients with nephrotic syndrome and its influence on the response of these patients to therapy [29–31]. Antioxidants can neutralize the negative effects of free radicals and reactive oxygen species (ROS) and reduce the effects of oxidative stress. Human saliva is rich in antioxidant compounds that comprise enzymes (e.g., peroxidase) and small molecules (e.g., uric acid). Salivary peroxidase is secreted by the acinar cells of salivary glands and catalyzes the oxidation of thiocyanate ions by hydrogen peroxide, thus preventing toxic accumulation of H_2_O_2_ [32]. Salivary peroxidase was suggested to be one of the salivary biomarkers in children with chronic kidney disease, but its level did not differ from that of the controls [33]. Our data also did not reveal any significant difference in SPO levels between all children with nephrotic syndrome and the healthy controls. However, the participants in the relapse phase of the disease revealed a significantly higher SPO level than those in the remission phase and the controls.

Serum uric acid is associated with many systemic conditions, including kidney and metabolic disorders. High uric acid levels have been found to be associated with a significant rapid decline in eGFR and can be considered a potential modifiable factor of chronic kidney disease progression [34]. In addition, uric acid is known as a scavenger of oxyradicals, a chelator of metal ions and an important antioxidant in plasma. Moreover, a correlation between uric acid concentrations in the saliva and plasma was noticed, and thus salivary uric acid can be used for monitoring the status of hyperuricemia [35]. We obtained a significantly higher concentration of salivary uric acid in iNS participants than in the controls, and some were higher in children with relapse than in those in the remission phase of the disease.

The elevated level of salivary urea was observed in patients with chronic kidney disease and was correlated with its blood content [36]. Our data showed only a trend of such a relationship. However, we noticed a significantly higher concentration of urea in the saliva of the iNS children than in the healthy children, and there was no difference between the patients with relapse and those in remission.

Patients with nephrotic syndrome can exhibit calcium homeostasis disturbance, which is assigned to the loss of various plasma proteins and minerals in urine and steroid therapy [37]. Significantly lower total calcium content in the serum was found in pediatric patients with steroid-resistant nephrotic syndrome compared with the controls, which, in addition, was lower in the relapse phase than in the complete remission phase [38]. Our results did not reveal any significant difference in salivary calcium between the iNS children and the controls or between patients with relapse and those in the remission phase of the disease.

A study on serum magnesium showed a significantly lower level in nephrotic syndrome patients than in controls. It has been deduced that the measurement of serum magnesium in children with acute nephropathy could be useful for early diagnosis and improving therapy [39]. Similarly, our results showed a significantly lower salivary magnesium concentration in the iNS children than in the controls and a lower magnesium level in patients with relapse than in those in remission.

An elevated serum phosphate level in nephrotic syndrome was found to be associated with the severity of the disease regardless of eGFR value and age [40]. However, no significant difference in serum phosphorus was found between patients with steroid-resistant nephrotic syndrome and the controls or between patients in complete remission and those with relapse [38]. Our data did not reveal any significant difference in the content of inorganic phosphate in the saliva between the iNS and healthy participants or in relation to the phase of the disease.

The sources of fluoride intake are diet and the fluoride compounds used for caries prevention, such as fluoridated water, fluoride supplements, and fluoridated toothpaste and mouth rinses when swallowed. The elimination of absorbed fluoride occurs almost exclusively through the kidney [41]. The excretion of fluoride is lower if the patient suffers from chronic renal failure, which leads to higher concentrations of fluoride in the serum and bone [42]. All participants in this study consumed drinking water with a low natural content of fluoride (< 0.3 ppm), and similar proportions of iNS and healthy participants declared the use of fluoridated dentifrice and professional topical application of fluoride specimens. We found a significantly lower concentration of fluoride in the saliva in the iNS group than in the controls.

However, despite the significant differences in mean magnesium, fluoride, urea and uric acid levels between the iNS and healthy participants, ROC curve analysis revealed that the discriminatory power of these variables for the differentiation of children with and without disease was weak. Similarly, salivary levels of peroxidase and magnesium were weak tests to identify iNS patients in the relapse phase of the disease. This finding indicates that the studied salivary parameters cannot be used as markers accurately differentiating iNS and healthy participants and those with relapse of the disease.

This study has some limitations. We evaluated only selected salivary parameters and thus could not fully characterize all potential changes in the salivary components. Moreover, we did not compare the same studied components in the saliva and the blood in all participants. The strength of the study was that the iNS and healthy participants subjected to the investigation did not differ significantly in the number of caries-affected primary and permanent teeth and gingival condition, which potentially could affect the studied salivary components.

Future long-term prospective investigations are needed to elucidate the possible impact of the disease course and treatment on the levels of salivary constituents in iNS patients.

## Conclusions

Within the limitations of the study, it may be stated that the levels of urea, uric acid, magnesium and fluoride in saliva were associated with disturbances occurring in the course of idiopathic steroid-sensitive nephrotic syndrome. Salivary levels of peroxidase and magnesium can be related to the phase of the disease. However, their measurements cannot serve as a noninvasive tool in diagnosing iNS and the phase of the disease.

## Abbreviations

DMFT: Decay, missing and filled permanent teeth
dmft: Decay, missing and filled primary teeth
API: Approximal Plaque Index
GI: Gingival Index
eGFR: Estimated glomerular filtration rate
V: Salivary flow
BC: Buffer capacity
P: Total protein
Amy: α-Amylase
SPO: Salivary peroxidase
Ca: Calcium
Mg: Magnesium
iPh: Inorganic phosphate
F: Fluoride
U: Urea
UA: Uric acid
WHO: World Health Organization
CKD: Chronic kidney disease
iNS: Idiopathic nephrotic syndrome
ROC: Receiver operating characteristic
AUC: The area under the ROC curve

## Declarations

### Availability of data and materials

The datasets used and analyzed during the current study are available from the corresponding author on request.
